# Pleiotropic Pharmacological Actions of Capsazepine, a Synthetic Analogue of Capsaicin, against Various Cancers and Inflammatory Diseases

**DOI:** 10.3390/molecules24050995

**Published:** 2019-03-12

**Authors:** Min Hee Yang, Sang Hoon Jung, Gautam Sethi, Kwang Seok Ahn

**Affiliations:** 1 KHU-KIST Department of Converging Science and Technology, Kyung Hee University, Seoul 02447, Korea; didmini@naver.com (M.H.Y.); shjung507@gmail.com (S.H.J.); 2 Department of Pharmacology, Yong Loo Lin School of Medicine, National University of Singapore, Singapore 117600, Singapore; 3 Department of Science in Korean Medicine, Kyung Hee University, 24 Kyungheedae-ro, Dongdaemun-gu, Seoul 02447, Korea; 4 Comorbidity Research Institute, College of Korean Medicine, Kyung Hee University, 24 Kyungheedae-ro, Dongdaemun-gu, Seoul 02447, Korea

**Keywords:** capsazepine, cancer, inflammatory diseases, ROS, TRPV1

## Abstract

Capsazepine is a synthetic analogue of capsaicin that can function as an antagonist of TRPV1. Capsazepine can exhibit diverse effects on cancer (prostate cancer, breast cancer, colorectal cancer, oral cancer, and osteosarcoma) growth and survival, and can be therapeutically used against other major disorders such as colitis, pancreatitis, malaria, and epilepsy. Capsazepine has been reported to exhibit pleiotropic anti-cancer effects against numerous tumor cell lines. Capsazepine can modulate Janus activated kinase (JAK)/signal transducer and activator of the transcription (STAT) pathway, intracellular Ca^2+^ concentration, and reactive oxygen species (ROS)-JNK-CCAAT/enhancer-binding protein homologous protein (CHOP) pathways. It can inhibit cell proliferation, metastasis, and induce apoptosis. Moreover, capsazepine can exert anti-inflammatory effects through the downregulation of lipopolysaccharide (LPS)-induced nuclear transcription factor-kappa B (NF-κB), as well as the blockage of activation of both transient receptor potential cation channel subfamily V member 1 (TRPV1) and transient receptor potential cation channel, subfamily A, and member 1 (TRPA1). This review briefly summarizes the diverse pharmacological actions of capsazepine against various cancers and inflammatory conditions.

## 1. Introduction

Capsaicin (8-Methyl-*N*-vanillyl-trans-6-nonenamide) is the commonly found pungent ingredient in hot chili peppers [1,2]. Capsaicin can act as a pharmacological agent that can regulate inflammation and pain using specific receptors of afferent sensory neurons [3]. The transient receptor potential vanilloid type 1 (TRPV1) channel can be activated by capsaicin [1]. TRPV1 is a ligand-gated non-selective, cation channel, and it was first reported in sensory neurons such as dorsal root ganglion (DRG) [4]. As soon as the TRPV1 channel is activated, uptake of calcium (Ca^2+^) ion is rapidly increased [5]. Ca^2+^ plays an important role in diverse signal transduction pathways [5], including cell proliferation, cell death, neural excitation, neurotransmitter release, etc.

**Figure 1:**
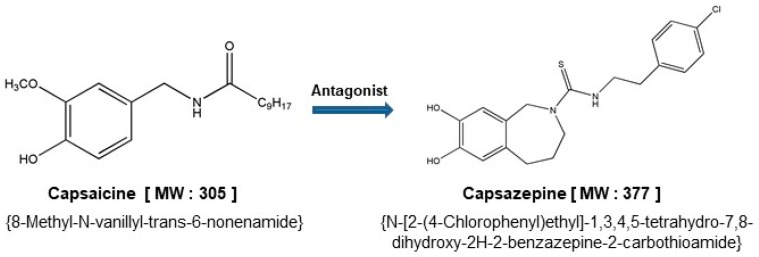
The chemical structures of capsaicin and capsazepine.

**Figure 2:**
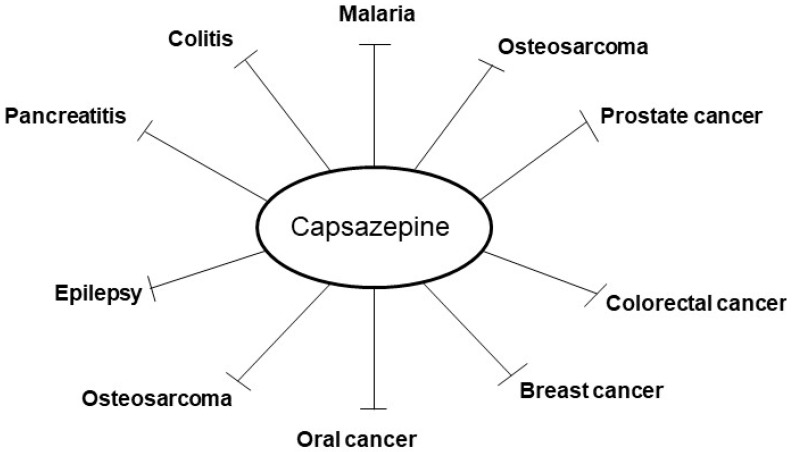
Pharmacological properties of capsazepine.

Capsazepine(*N*-[2-(4-Chlorophenyl)ethyl]-1,3,4,5-tetrahydro-7,8-dihydroxy-2*H*-2-benzazepine-2 carbothioamide) is a synthetic analogue of capsaicin [1]. It was first discovered and characterized by the Sandoz (now Novartis) [1], and it was modified on the chemical backbone of capsaicin [6], (Figure 1). Interestingly, capsazepine (10 µM) can also reversibly reduce the response to capsaicin (500 nM) of voltage-clamped DRG neurons in rats [1]. Moreover, capsazepine can act as a potent blocker of TRPV1 channels. It can bind to the pores of transmembrane domain on TRPV1 channel and can interact with all monomers residues of this channel [3]. Capsazepine can also exhibit several pharmacological effects via blocking TRPV1 channel and thereby suppressing the influx of Ca^2+^ [5]. It can thus be effectively used for the prevention and treatment of various cancers and inflammatory conditions, although its clinical use has been hampered, owing to its poor pharmacokinetic properties (Figure 2).

Additionally, capsazepine has been also reported to target various other receptors including other TRP channels such as TRPV4 and TRPM8 [7,8,9]. It can also block nicotinic acetylcholine receptors and voltage-activated calcium channels in rats [7,8]. Interestingly, Docherty et al. reported that capsazepine can mediate human hyperpolarization-activated cyclic nucleotide-gated two and four channels and inhibit currents in the HEK293 cells concentration dependently [9]. This finding can also partly explain the reported anti-nociceptive effects of capsazepine [9].

## 2. Pharmacological Actions of Capsazepine in Tumor Cell Lines

### 2.1. Anti-Cancer Effects of Capsazepine In Vitro

**Figure 3:**
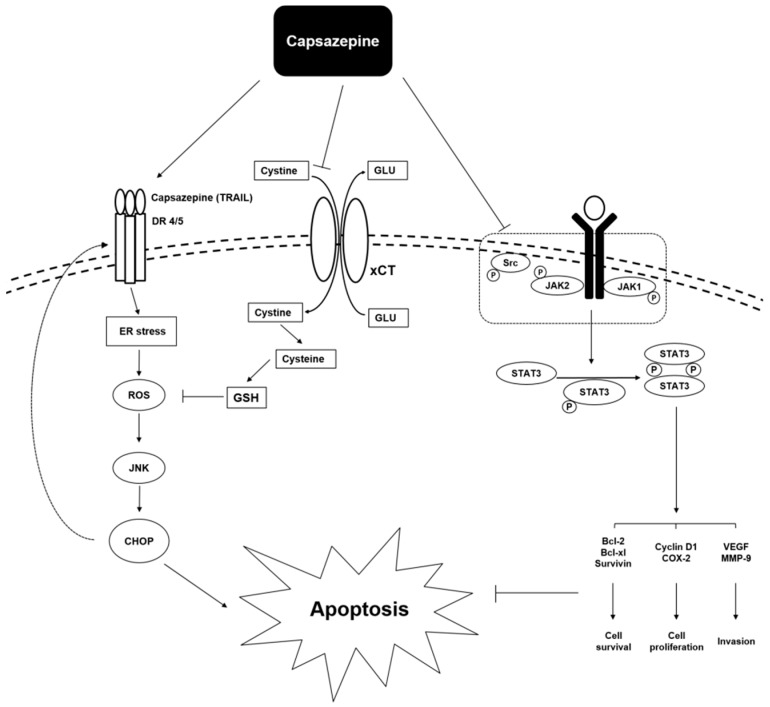
Potential mechanisms underlying reported anti-cancer effects of capsazepine.

Several compounds derived from Mother Nature can function as potent anti-cancer agents that can abrogate the process of tumorigenesis [10,11,12,13,14,15,16,17,18,19,20,21,22,23,24,25,26]. Capsazepine has been reported to exert significant anti-proliferative effects against multiple tumor types *in vitro*, as summarized in Table 1. The mechanisms underlying the anti-cancer/growth inhibitory effects include the inhibition of activation of Janus activated kinase (JAK)/signal transducer and activator of transcription (STAT) pathway, calcium ion influx, ROS-JNK-CHOP pathway, and modulation of other important signal transduction pathways (Figure 3).

#### 2.1.1. Prostate Cancer

Signal transducer and activator of transcription (STAT) proteins activation associated with cell proliferation, survival, and angiogenesis [27,31,32,33,34,35,36,37,38,39,40]. STAT3 is frequently hyper-activated in tumor cells and regulates the expression of oncogenic genes [31]. Capsazepine was found to induce substantial apoptosis in DU145 and PC-3 prostate cells by inhibiting STAT3 activation [27]. The suppression of STAT3 was caused through the inhibition of upstream Janus activated kinase-1, 2 (JAK1, JAK2), and c-Src kinases. Moreover, capsazepine induced the expression of PTPε both protein and mRNA levels that may mediate the STAT3 inhibitory effects of the drug [27]. Capsazepine also decreases the expression of various oncogenic proteins, invasion, and promoted apoptosis in prostate cancer [27].

While capsazepine has been known to be a potent blocker of the TRPV1 channel. Huang et al. reported that capsazepine can exhibit anticancer effects in prostate cancer by inducing intracellular Ca^2+^ concentration. There are two different ways to store Ca^2+^. For example, IP_3_-sensitive Ca^2+^ stores release Ca^2+^ into the cytosol when cells are stimulated by an endogenous agent, whereas IP_3_-insensitive Ca^2+^ stores can release Ca^2+^ into the cytosol when cells are stimulated by the exogenous agent [4]. Human PC-3 cells can store Ca^2+^ in the endoplasmic reticulum [4]. Capsazepine induced intracellular Ca^2+^ concentration by Ca^2+^ influx, and thereby releasing Ca^2+^ from the endoplasmic reticulum [4]. Interestingly, capsazepine causes the release of Ca^2+^ from the endoplasmic reticulum in a phospholipase C independent manner as the U73122, an inhibitor of phospholipase C, treatment did not significantly effect capsazepine-induced Ca^2+^ release [4].

#### 2.1.2. Breast Cancer

System x_c_^−^ (xCT), the functional unit of cys-tine/glutamate antiporter, has been found to be elevated in many tumor types in response to high ROS concentrations [28]. When this antiporter is upregulated, it can promote cell survival by inducing cysteine uptake and promoting glutathione (GSH) production [28]. High glutamate released by System x_c_^−^ (xCT) has been associated with cancer-induced bone pain (CIBP) during distal breast metastasis [28]. The exchange of cystine for glutamate generally occurs at a stoichiometric ratio of 1:1 induced by the intracellular concentration of glutamate [28]. Therefore, the inhibition of System x_c_^−^ (xCT) can induce the downregulation of glutamate release, and thus reduce mechanical hyperalgesia associated with CIBP [28]. Capsazepine was found to significantly inhibit System x_c_^−^ (xCT) by blocking the uptake of cysteine [28]. Capsazepine was also found to induce ROS production, which led to a substantial programmed cell death in MDA-MB-231 cells [28].

#### 2.1.3. Colorectal Cancer

TNF-related apoptosis-inducing ligand (TRAIL) has the role of anti-cancer effects [5]. TRAIL can bind to the death receptors and activate the extrinsic apoptotic cell death pathway [5,41,42,43,44]. TRAIL can induce cancer apoptosis by increasing the activation of death receptors DR4 and DR5. DR induction has been related to the increased activation of CCAAT/enhancer-binding protein homologous protein (CHOP), ROS production, as well as to the augmented JNK phosphorylation [45,46]. Interestingly, capsazepine was found to induce TRAIL receptor expression by upregulating both DR4 and DR5 receptors through JNK activation in colorectal HCT116 cells [5]. It also required ROS and CHOP to exert these effects [5]. Capsazepine also decreased the expression of cell survival proteins and increases the pro-apoptotic proteins [5].

#### 2.1.4. Oral Cancer

Gonzales et al. reported that capsazepine can exhibit both cytotoxic and anti-tumor effects in oral squamous cell carcinoma (OSCC) [29]. These effects were associated with the production of ROS independently of its action on the TRPV1 channel [29]. ROS can regulate the activation of various signaling molecules including NF-κB, STAT3, JNK, hypoxia-inducible factor-1α, kinases, growth factors, cytokines and other proteins, and enzymes [29,35,38,47,48,49,50,51,52]. It has been closely linked to cell proliferation, survival, invasion, and metastasis of cancer [48,53]. It is well known that cancer cells undergo oxidative stress due to increased metabolic activity resulting in a subtle balance between ROS levels and cellular antioxidant capabilities. When ROS levels are increased above basal level, the subtle balance may be disrupted and thus trigger ROS induced apoptosis. Vanilloids such as capsazepine have been found to increase ROS and thus alter the balance between normal ROS contents and cellular antioxidant capabilities [29,54,55]. Capsazepine was also observed to augment apoptosis in a concentration-dependent manner in SCC4, SCC25, and HSC3 cells [29].

#### 2.1.5. Osteosarcoma

Capsazepine can also exert potent anti-cancer effects on MG63 osteosarcoma cells [30]. Capsazepine can induce intracellular Ca^2+^ increase by causing extracellular Ca^2+^ influx [30]. Moreover, capsazepine can cause intracellular Ca^2+^ release from endoplasmic reticulum via a phospholipase C-independent manner [30]. It was also noted to attenuate cell proliferation in a concentration dependent manner [30]. The multiple oncogenic targets modulated upon capsazepine treatment are briefly summarized in Figure 3.

### 2.2. Anti-Cancer Effects of Capsazepine In Vivo

#### 2.2.1. Prostate Cancer

Capsazepine has been reported to exhibit anti-cancer effects in prostate cancer in preclinical settings [27]. Capsazepine administered at doses of 1 mg/kg and 5 mg/kg three times a week for up to 20 days abrogated tumor growth in the xenograft prostate cancer mouse model [27]. Additionally, capsazepine treatment caused reduction in phosphorylation of STAT3 and increased PTPε protein levels in tumor tissues [27].

#### 2.2.2. Breast Cancer

Capsazepine can regulate System x_c_^−^ activity under *in vivo* conditions as well [28]. MDA-MB-231 grafted BALB/c nude mice was treated with high (10 mg/kg) and low (5 mg/kg) doses of capsazepine for three days/week and it was found to delay the CIBP-induced nociceptive behaviors [28].

#### 2.2.3. Oral Cancer

Capsazepine treatment in oral squamous cell carcinoma (OSCC) xenograft mouse model was observed to attenuate tumor growth [29]. HSC3, SCC4, and SCC25 xenografts were treated with 0.02, 0.04 mg capsazepine for 12, 16, or 18 days, respectively. Anti-tumor effects of capsazepine has no adverse effects on non-malignant tissues *in vivo* [29] (Table 2).

## 3. Effects of Capsazepine on Inflammatory Conditions

Lipopolysaccharide (LPS) can interact with Toll-like receptor 4 (TLR4), leading to the activation of nuclear transcription factor-kappa B (NF-κB), a transcription factor that plays an important role in both inflammation and cancer [56,57,58,59,60,61,62,63,64,65,66,67]. NF-κB can initiate the transcription of inducible nitric oxide synthase (iNOS), tumor necrosis factor-α(TNF-α), interleukin-6 (IL-6), and other pro-inflammatory mediators [68]. Nitric oxide (NO) is one of the key products generated during an inflammatory response [69,70]. Capsazepine can downregulate NO production by attenuating iNOS mRNA expression in LPS-stimulated RAW264.7 macrophages [70]. Capsazepine was also found to abrogate LPS-induced NF-κB activation and it was noted that these inhibitory effects were mediated via its antioxidant activity [70].

Capsazepine is an effective blocker at TRPV1 in human, rat, and guinea pig. Capsazepine can block the TRPV1 responses in response to low pH and heat in human and guinea pig with a better efficacy than in rat [71]. Additionally, capsazepine has been reported to reduce both inflammatory and neuropathic mechanical hyperalgesia in guinea pigs, but not in rats [72]

### 3.1. Colitis

**Figure 4:**
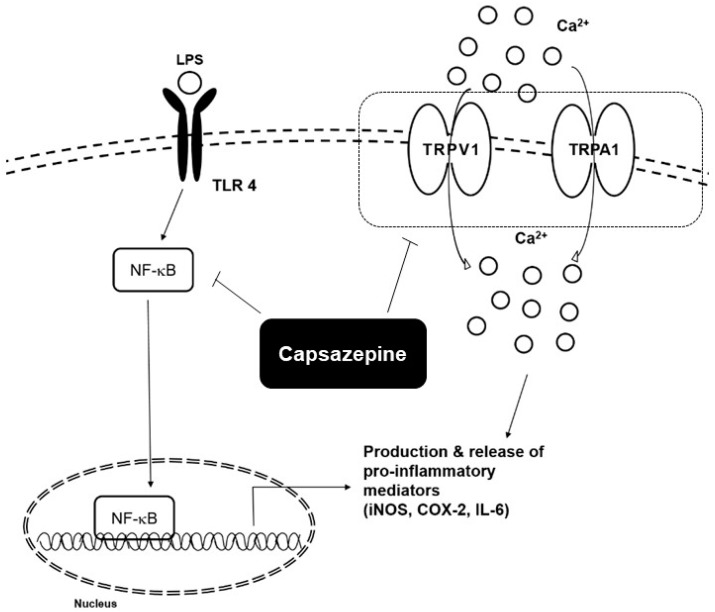
Potential mechanisms regulating anti-inflammatory effects of capsazepine.

Sensory neurons have two major polymodal ion channel receptors, TRPV1 and transient receptor potential ankyrin 1 (TRPA1) [73]. Sensitization of both TRPA1 and TRPV1 can lead to hyperalgesia and both channels can also exert neurogenic inflammatory effects [73]. TRPA1 was found in DRG and has an important role in peripheral pain [73]. TRPA1 can also exert anti-inflammatory and anti-nociceptive effects similar to TRPV1 [73]. Kistner et al. found that capsazepine can also exhibit inhibitory effects on colitis via the modulation of TRPA1 [73]. They demonstrated this hypothesis by using capsazepine-induced calcium transients in human TRPA1-expressing HEK293t cells and mice [73]. The diverse pro-inflammatory mediators affected by capsazepine treatment are depicted in Figure 4 (Table 3).

Attenuation of experimental colitis by capsazepine has been attributed to its antagonistic effects on TRPV1channel, and were also found to be associated with the inhibition of neurogenic inflammation [74]. For example, repeated capsazepine administration can attenuated trinitrobenzene sulfonic acid (TNBS)-induced colitis in rats [74]. Rats were treated with 37.7 × 10^−5^ mg/kg/day of capsazepine enema for six days [74]. Capsazepine was found to downregulate macroscopic damage score (MDS) and MPO scores [74]. Similarly, capsazepine can prevent intestinal inflammation in dextran sulphate sodium (DSS)-induced colitis [75]. Sprague-Dawley rats were treated with 0.1 mg/kg/day for six days [75]. Capsazepine significantly decreased the levels of disease activity index (DAI), myeloperoxidase (MPO) activity in DSS-induced colitis [75].

### 3.2. Pancreatitis

TRPV1 activation was also found to be involved in acute pancreatitis. Wick et al. reported that the sensory nerves that stimulate pancreas can release TRPV1, substance P (SP), and CGRP in dorsal horn caused during the nociception process [81]. Antagonism of TRPV1, SP, and CGRP receptors can inhibit pancreatitis pain [81]. Additionally, pancreaticobiliary duct obstruction may cause an increase in the pancreatic leukotriene B_4_ (LTB_4_) concentrations [76]. It can thus mediate TRPV1 activation and causes acute pancreatitis. Rats were pre-treated with capsazepine 37.7 × 10^−3^ mg/kg sc 30 min before surgery [76]. Capsazepine caused a downregulation of various inflammatory parameters such as myeloperoxidase (MPO) activity, pancreatic edema, and histological damage in leukotriene B_4_ (LTB_4_)-induced pancreatitis [76].

### 3.3. Malaria

Malaria is an infectious disease caused by the bite of infectious mosquitoes and the outcome of infection depends on the host’s innate immune response [82]. White et al. investigated the role of TRPV1 in malaria for the first time and employed C57BL/6 mice treated with capsazepine 0.05 mg/kg/day for six days [82]. They found that capsazepine was able to regulate the innate immune response to malaria in mice infected with *Plasmodium berghei* ANKA [82].

### 3.4. Epilepsy

Calcium ion accumulation in hippocampal neurons is a major contributor to epilepsy [80]. Ghazizadeh et al. and Naziroglu et al. investigated that epilepsy effects on oxidative stress [83,84]. They found that Ca^2+^ signaling and the apoptosis in pentylentetrazol (PTZ)-induced hippocampal injury in rats. Shirazi et al. reported that TRPV1 receptors are important for PTZ and amygdala-induced kindling in rats [85]. TRPV1 antagonist, capsazepine can modulate epileptiform activity by anti-convulsant properties [85]. During epilepsy induction, intracellular calcium ion concentration was found to be increased [85]. Capsazepine caused a decrease in intracellular Ca^2+^ concentration [85]. There are many studies anti-epileptic effect of capsazepine [6,27,80,86,87]. Gonzalez-Reyes et al. reported that the capsazepine administration can suppress 4-AP induced ictal activity and propagation of seizure activity *in vitro* (10–100 µM) and *in vivo* (50 mg/kg s.c.) [80]. In addition, capsazepine can act directly on the axons through the blood brain barrier [80]. Nazıroğlu et al. has also shown that capsaicin-induced TRPV1 sensitization can cause Ca^2+^ elevation, thereby increasing apoptosis and epileptic seizures [80]. These processes were reduced by capsazepine (0.1 mM) treatment [87]. Additionally, capsazepine can potentiate the anti-nociceptive effects of morphine in mice [79]. Morphine treatment can induce TRPV1 expression in the DRG, spinal cord upon repeated exposure [79]. Interestingly, TRPV1 antagonists can be used effectively as pharmacological agents against morphine treatment. Santos et al. found that capsazepine treatment can lead to an inhibitory avoidance, thereby leading to a decrease in the rat elevated plus-maze test and thus indicating that TRPV1 may have a key role in regulating anxiety [88]. Similarly, a decreased expression of TRPV1 channels and inhibitory avoidance behavior was observed in rats that received capsazepine in the elevated plus-maze test [69] (Table 4).

### 3.5. Neurogenic Inflammation

Capsazepine can inhibit neurogenic inflammation mediated by TRPV1 [89,90]. Inflammatory responses caused by the release of inflammatory mediators such as neuropeptide calcitonin gene-related peptide (CGRP) and substance P (SP) from primary afferent nerve terminals are referred to as neurogenic inflammation [89,91]. Inflammatory neuropeptides release by antidromic activation of afferent nociceptors and dorsal root reflexes (DRRs) play a key role in this process [91]. Flores et al. reported that capsazepine (300 μL) abolished the capsaicin-evoked release of immunoreactive CGRP (iCGRP) in Sprague-dawley rats buccal mucosa [92]. Moreover, the neurosecretion of capsaicin-evoked iCGRP via the vanilloid receptor mediated mechanism [92].

Further, capsazepine can inhibit H_2_S-induced neurogenic inflammation [89,90,93]. Hydrogen sulfide (H_2_S) is a mediator of diverse biological effects [89]. It also contributes to local and systemic inflammation [94]. Sodium hydrogen sulfide (NaHS) used as a donor of H_2_S and induces sensory nerve activation in the guinea pig airways [89]. Capsazepine can abrogate NaHS evoked neuropeptide release through desensitization of TRPV1 [89]. Bhatia et al. noted that capsazepine pretreatment (15 mg/kg) in mice can protect H_2_S-inducing lung inflammation [90]. Additionally, they found that H_2_S is located upstream of TRPV1 activation, and can regulate the release of sensory neuropeptides in sepsis [93].

## 4. Conclusions and Future Perspectives

In this article, we have briefly reviewed diverse pharmacological actions of capsazepine *in vitro* and *in vivo*. Capsazepine can exert therapeutic effects against various malignancies and inflammatory disorders. It can suppress proliferation and metastasis, induce apoptosis by modulating several oncogenic signaling pathways, and thereby exert its anti-tumoral effects in different cancers. Moreover, capsazepine can reduce the levels of inflammatory mediators such as DAI, and MPO activity, however, the concentrations at which it can exert these pleiotropic anti-tumoral/anti-inflammatory effects may vary depending on the cell types and *in vivo* model systems used for investigation. Additional studies are required to elucidate the unmet potential of capsazepine in suitable animal models and clinical settings.

## Figures and Tables

**Table 1:** Anti-cancer effects of capsazepine *in vitro.*

Origin	Cell Lines	Concentrations	Molecular Targets	Mechanism of Actions	Ref.
Prostate	DU145	1, 2.5, 5 μM for 6, 24 h	STAT3, JAK↓	Apoptosis↑	[27]
	LNCaP				[27]
	PC-3	200 μM from for 5 h	Intracellular Ca^2+^ concentration↑	Apoptosis↑	[4]
Breast	MDA-MB-231	25 μM for 48 h	System x_c_^−^ (xCT), cystine	ROS↑ Apoptosis↑	[28]
Colon	HCT166	10, 30 μM for 6, 24 h	ROS, JNK, CHOP	Apoptosis↑	[5]
Oral	SCC4	30, 60, 90 μM for 24 h	ROS	cell proliferation↓ Apoptosis↑	[29]
	SCC25				
	HSC3				
Bone	MG63	50, 100, 150, 200 μM for 4 h	Intracellular Ca^2+^ concentration↑	Tumor cell multiplication↓	[30]

**Table 2:** Anti-cancer effects of Capsazepine on animal studies.

Disease	Animal Model	Dosage	Outcome	Ref.
Prostate cancer	mice	1, 5 mg/kg/day for 20 days	Tumor growth↓	[27].
			STAT3↓	
Breast cancer	mice	10, 5 mg/kg/day for 36 days	CIBP-induced nociceptive behaviors	[28]
Oral cancer	mice	0.02, 0.04 mg/day for 12, 16 and 18 days	Tumor growth↓	[29]

**Table 3:** Anti-inflammatory Effects of capsazepine *in vitro*.

Origin	Cell Lines	Concentrations	Molecular Targets	Mechanism of Actions	Ref.
Macrophage	RAW264.7	1, 5, 10 μM for 6 h	NF-κB	Immune response↑	[70]
Kidney	HEK293t	10 μM for 10 s	TRPA1	Inflammation↓	[73]
Hippocampus	Hippocampal ca1 pyramidal cells	10, 100 μM for 20 min	TRPV1	Apoptosis↑, cell proliferation↓	[76]

**Table 4:** Anti-inflammatory effects of capsazepine in preclinical disease models.

Disease	Animal Model	Dosage	Outcome	Ref.
Colitis	Rat	37.7 × 10^−5^ mg/kg/day for 6 days	Inflammatory parameter↓	[74]
	Rat	0.1 mg/kg/day for 6 days	DAI, MPO activity↓	[74]
	Rat	1 mg/kg/day for 7 days	Inflammatory parameter↓	[73]
Pancreatitis	Rat	37.7 × 10^−3^ mg/kg for 30 min before surgery	Inflammatory parameter↓	[76]
Malaria	mice	0.05 mg/kg/day for 6 days	Immune response↑	[77]
Epilepsy	rat	1, 3, 10 mg/kg/day for 7 days	seizure severity↓	[78]
	mice	5 mg/kg/test	antinociceptive effects	[79]
	mice	50 mg/kg	4-AP(4-aminopyridine)-induced epileptic status activity↓	[80]

## Abbreviations

TRPV1	Transient receptor potential vanilloid type 1
DRG	Dorsal root ganglion
STAT	Signal transducer and activator of transcription
JAK1, JAK2	Janus activated kinase-1, 2
GSH	Glutathione
CIBP	Cancer-induced bone pain
ROS	Reactive oxygen species
CHOP	CCAAT/enhancer-binding protein homologous protein
LPS	Lipopolysaccharide
NF-κB	Nuclear transcription factor-kappa B
OSCC	Oral squamous cell carcinoma
TLR4	Toll-like receptor 4
iNOS	Inducible nitric oxide synthase
TNF-α	Tumor necrosis factor-α
IL-6	Interleukin-6
NO	Nitric oxide
TRPA1	Transient receptor potential ankyrin 1
TNBS	Trinitrobenzene sulfonic acid
MDS	Macroscopic damage score
MPO	Myeloperoxidase
DSS	Dextran sulphate sodium
LTB_4_	Leukotriene B_4_
PTZ	Pentylentetrazol
CGRP	Calcitonin gene-related peptide
